# Efficacy and safety of using aminocaproic acid and tranexamic acid during the perioperative period for treating trochanteric fractures in elderly femurs

**DOI:** 10.1186/s12891-023-06627-z

**Published:** 2023-07-03

**Authors:** Alimujiang Yusufu, Abuduwupuer Haibier, Zheng Ren, Qi Qin, Ziyi Zhang, Yuan Zhou, Jian Ran

**Affiliations:** 1 Department of Orthopedics of Trauma, Sixth Affiliated Hospital of Xinjiang Medical University, Orthopaedic Hospital of Xinjiang Uygur Autonomous Region, No.39 Wuxing Road, Urumqi, People’s Republic of China; 2 Xinjiang Medical University, Urumqi, Xinjiang Uygur Autonomous Region People’s Republic of China

**Keywords:** Proximal femoral fracture, Tranexamic acid, Aminohexanic acid, Blood loss

## Abstract

**Background:**

Tranexamic acid (TXA) has long been the antifibrinolytic hemostatic drug of choice for orthopedic surgery. In recent years, the hemostatic effect of epsilon aminocaproic acid (EACA) has gradually been recognized by orthopedic surgeons and has begun to be used in hip and knee arthroplasty with little mention of the comparison of these two drugs; Therefore, this study compared the efficacy and safety of EACA and TXA in the perioperative period of elderly patients with trochanteric fractures to verify whether EAC could be a "qualified alternative" to TXA and to provide theoretical support for the clinical application of TXA.

**Methods:**

Two hundred and forty-three patients who received proximal femoral nail antirotation (PFNA) for trochanteric fractures from January 2021 to March 2022 at our institution were included and divided into the EACA group (*n* = 146) and the TXA group. (*n* = 97) determined by the drugs used in the perioperative period The main observations were blood loss and blood transfusion.The second second outcome was blood routine, coagulation, Hospital complications and complications after discharge.

**Results:**

The perioperative EACA patients had significantly lower significant blood loss (DBL) than the TXA group (*p* < 0.0001) and statistically significant lower C-reactive protein in the EACA group than in the TXA group on postoperative day 1 (*p* = 0.022). Patients on perioperative TXA had better postoperative day one (*p* = 0.002) and postoperative day five erythrocyte width than the EACA group (*p* = 0.004). However, there was no statistically significant difference between the two groups in the remaining indicators in both drugs: blood items, coagulation indicators, blood loss, blood transfusion, length of hospital(LOH), total hospital expense, and postoperative complications (*p* > 0.05).

**Conclusion:**

The hemostatic effects and safety of EACA and TXA in the perioperative application of trochanteric fractures in the elderly are essentially similar, and EACA can be considered for use as an alternative to TXA, increasing the flexibility of physicians to use it in the clinical setting. However, the limited sample size included necessitated a high-quality, large sample of clinical studies and long-term follow-up.

## Background

Proximal femoral fractures are one of the most common types of hip fractures in the elderly population [1]. Due to chronic underlying diseases such as osteoporosis in the elderly, proximal femoral fractures have a high mortality rate and are therefore also referred to as the "last fracture of life". In elderly patients, due to their poor physical condition and complex and variable disease, early surgery can restore the muscle strength of the lower limbs through early functional exercise, which can lead to a rapid recovery and reduce the probability of complications [2, 3]. Intramedullary fixation surgery has become the treatment of choice for intertrochanteric femoral fractures [4]. Although PFNA has the advantages of a short operative time and minimal medically induced trauma, intraoperative marrow expansion can lead to increased occult blood loss, and it is estimated that patients with femoral intertrochanteric fractures have an average perioperative blood loss of up to 2100 m [5], and the occurrence of anemia is more common. Studies have reported perioperative transfusion requirements for intertrochanteric femur fractures to range from 30% to 84.6%, depending on the type of fracture and age [5–8]. Blood transfusions are associated with many adverse events, including hemolytic transfusion reactions, postoperative infections, longer hospital stays, and costly treatment [9]. Therefore, there is an urgent need to establish an optimal strategy to reduce blood loss to reduce the cost of surgery and improve patient prognosis.

A variety of hematoprotective measures have been used clinically, including spinal anesthesia, tourniquets, and reinfusion drainage, but each of these methods has different limitations [10]. However, the use of antifibrinolytics has been shown to be a more effective perioperative hematologic management measure than previous strategies [11]. TXA and EACA are the most common antifibrinolytic agents with essentially similar antifibrinolytic mechanisms [12]. Previous studies have shown [13] that both antifibrinolytic agents are associated with a significant reduction in perioperative blood loss and transfusion requirements in orthopedic surgery. To date, very few studies have reported the efficacy of aminocaproic acid for perioperative use in intertrochanteric fractures of the femur.

Therefore, we conducted a retrospective cohort study to investigate the efficacy of EACA versus TXA in terms of hemostatic effect and safety of PFNA in elderly intertrochanteric fractures to investigate whether EACA can be used as an alternative to TXA in such fractures, and to draw clinical conclusions and to promote it.

## Methods

### Patients and design

**Fig.1:**
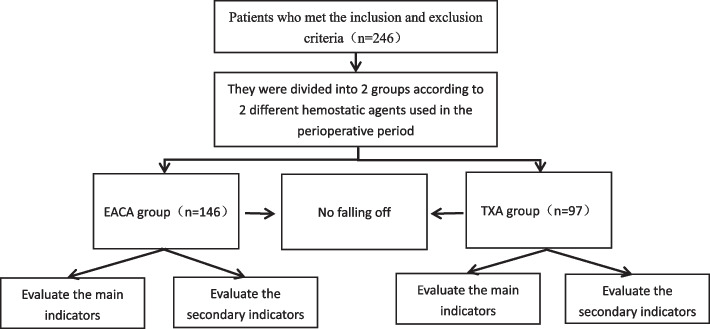
Patient flow chart

This is a retrospective study approved by the Ethics Committee of the Sixth Affiliated Hospital of Xinjiang Medical University, ethical approval number LFYLLSC20221018-01. All of the study subjects signed the informed consent form (Fig. 1).

**Fig. 2:**
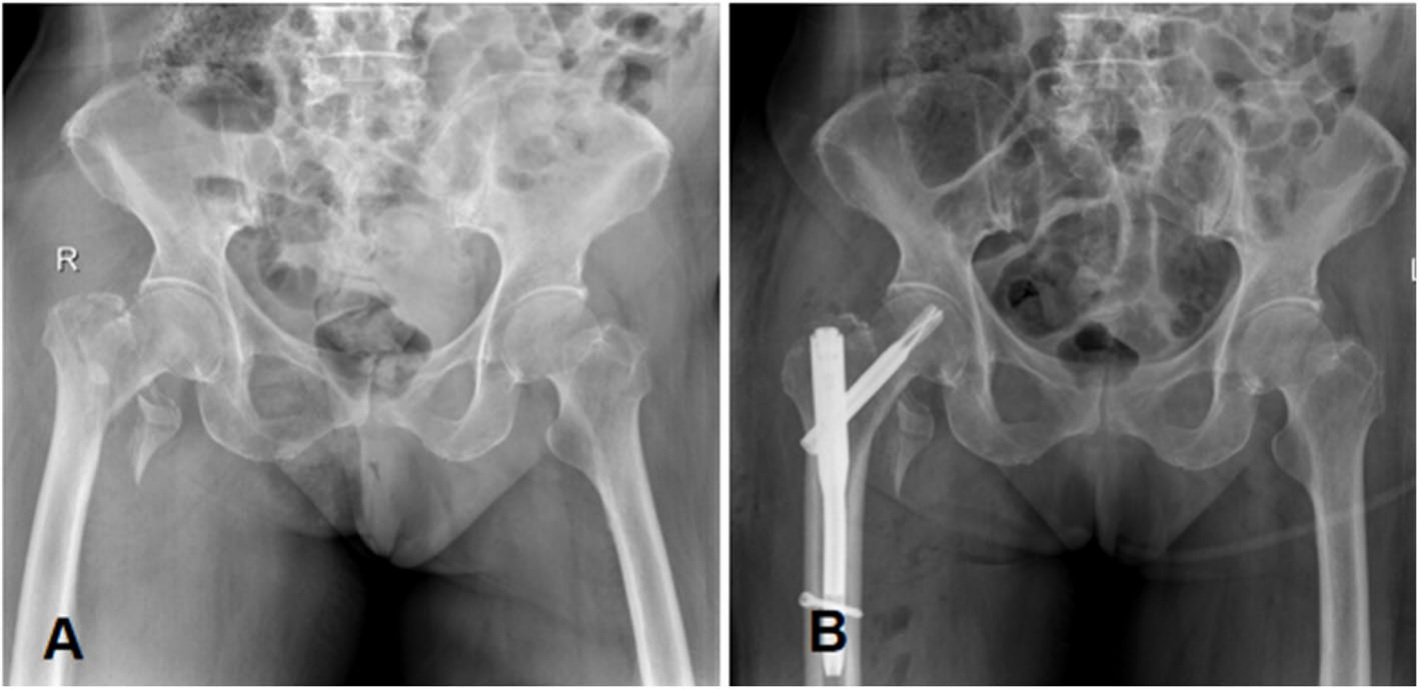
(**A**) pre-operative x-ray film of right femoral intertrochanteric fracture; (**B**) x-ray film after PFNA operation of right femoral intertrochanteric fracture

According to the recommendations in the literature [14], all TXA groups in our trial were given the standard dose of 1 g TXA i.v. TXA is at least 7 times higher than EACA on a molar basis and therefore requires less drug dose; second, several studies have shown that the optimal dose of EACA is 15 to 30 min before the incision and an additional dose (2 to 4 g) before incision. The drug dose was in accordance with the recommendations of the anesthesia team of the Sixth Affiliated Hospital of Xinjiang Medical University. Inclusion criteria: ① Diagnosis of intertrochanteric fracture by imaging examination (X-ray/CT). The presentation of a pertrochanteric femoral fracture of types 31-A1 to A3 according to the AO classification was defined as the primary inclusion criterion (Fig. 2). ②Age ≥ 70 years old ③No hematologic disorders ④Normal preoperative vascular ultrasound findings in both lower extremities ⑤Normal coagulation function and no long-term anticoagulant ⑥Complete medical records ⑦Patients who behaved autonomously and gave informed consent for the test.

### Surgical procedure and postoperative rehabilitation

After admission, the affected limb underwent continuous tibial tubercle bone traction and a routine preoperative examination. Patients with internal diseases underwent special internal examinations. In addition, anemia, electrolyte disorder, hypoproteinemia and diabetes were actively corrected before surgery. The repositioning is performed on a traction bed using epidural or general anesthesia and confirmed by fluoroscopy with a C-arm. A straight incision was made 5 cm at the tip of the great trochanter of the femur, and the hole at the tip of the large tuber was opened to expand the pulp and insert the guide needle. The main nail was placed after fluoroscopic confirmation by C-arm and the femoral neck screw and hip screw guide needle were monitored by the C-arm X-wire machine. The proximal anti-rotation screw was drilled first, then the distal compression screw was screwed, and finally the distal 2 interlocking screws were screwed, and the incision was closed layer by layer.

All patients were routinely given a 5% glucose injection (500 mL:25 mg, Sichuan Kelun Pharmaceutical Co., Ltd.) + sodium lactate Ringer injection (500 mL, Xinjiang Huashidan Pharmaceutical Co., Ltd.) toreplenish blood volume and maintain electrolyte balance within 4 h after the operation. The antithrombotic regimen was the same in both groups: all patients were given 4,100 IU of calcium nadroparin injection (0.4 mL:4100 IU, Nanjing Jianyou Biochemical Pharmaceutical Co., Ltd.) within 6 h after the operation, and 4100 IU nadroparin calcium was injected subcutaneously every 24 h until the patients were discharged from the hospital 2 weeks after surgery. If the patients experienced pain and discomfort, ibuprofen codeine extended-release tablets or intramuscular diazoxide were given. All patients were encouraged to perform equal length and knee flexion exercises of the ankle and quadriceps on the second day after the operation. On postoperative days 1, 3, 5, and 9, the competent physician cleans and changes the incision with iodophor and observes the recovery of the incision. Any abnormalities should be reported and treated promptly. Ultrasound examination of blood vessels of both lower extremities was performed on the 7th day after the operation, and stitches were routinely removed On postoperative day 12, but if the patient developed complications such as incision edge necrosis or fat liquefaction, the competent physician should report to the superior physician, and the stitches could not be removed and discharged until the incision healed completely. After discharge, the patient should be informed to come to the hospital for regular review of lower limb vascular ultrasound and coagulation function 1 or 2 months after surgery; during this period, the patient should be strictly bedridden and should not bear weight on the floor.

### Outcome measurements

Demographic characteristics and medical history of the patients were documented preoperatively.

The primary outcomes were blood loss and blood transfusion volume. The indicators of blood loss included total blood loss (TBL), dominant blood loss (DBL) and hidden blood loss (HBL). TBL = 1000 × total erythroid loss/average hematocrit. DBL = Intraoperative blood loss + postoperative drainage flow (all patients had no drain placed postoperatively). HBL = TBL-DBL + blood transfusion. Patient’s blood volume(PBV) [15] = K1 × (height 3) + K2 × (weight) + K3), male patients K1 = 0.3669, K2 = 0.03219 and K3 = 0.6041, while female patients K1 = 0.3561, K2 = 0.03308 and K3 = 0.1833 [16, 17]. Total red blood cell loss = PBV × (preoperative hematocrit-postoperative hematocrit). Mean hematocrit = (preoperative hematocrit + postoperative hematocrit)/2.

No postoperative drains were placed in all patients, so the drainage rate was 0. According to the bleeding amount in the suction storage tank and the gauze blood suction tank of the nurse under the operating table, there may be errors, but it is meaningful for the surgeon to quickly determine the amount of bleeding. Secondary outcomes included routine blood tests, C-reactive protein and coagulation function, LOH, operative time, total hospital expense, complete weight-bearing time and postoperative complications (thromboembolic adverse events, fatal events, and recurrent fractures). LOH was calculated from the first day of admission to the day of discharge. Total hospitalization expenses were based on the amount spent by the patient between admission and discharge.

### Statistical analysis

SPSS26.0 software was used to analyze the data. The results of all measured data in this study were expressed as mean ± standard deviation, and the classified data were expressed as percentags and frequencies. Because no significant deviation from normality was detected, t-tests were used to compare continuous variables between the two treatment groups (EACA and TXA), and chi-square tests or Fisher exact tests were used to compare categorical variables.

## Results

### Patient demographics

A total of 243 patients with trochanteric fractures (97 in TXA and 146 in EACA) were collected for this study. Baseline characteristics in both groups were comparable, as summarized in Table 1.

**Table 1:** Baseline characteristics

Index	EACA(*n* = 146)	TXA(*n* = 97)	t	*P*
Age(y, $y, \overline{X }\pm S$	76.55 ± 5.06	77.17 ± 5.48	0.380	0.369
Sex (n, Male/Female)	50/96	36/61	0.209	0.647
BMI $\left(\frac{\mathrm{kg}}{{\mathrm{m}}^{2}}, \overline{X }\pm S\right)$	23.36 ± 3.84	23.76 ± 4.03	0.787	0.432
Position(Left/Right)	67/79	46/51	0.055	0.815
Basic disease(n)				
Hypertension	74	42	1.274	0.259
Diabetes	45	25	0.724	0.395
Cardiovascular system	57	36	0.092	0.762
Nervous system	37	22	0.225	0.636
Respiratory system	14	8	0.127	0.721
Osteoporosis	27	12	1.621	0.203
Preoperative values				
Red blood cell(1012/L)	3.83 ± 0.65	3.95 ± 0.71	1.321	0.188
Platelets(1012/L)	209.96 ± 76.51	214.54 ± 84.96	0.437	0.662
Red blood cell width(SD)	41.93 ± 10.53	41.87 ± 9.60	-0.051	0.960
Hemoglobin(g/L)	116.94 ± 20.44	121.35 ± 20.73	1.636	0.103
Hematocrit(%)	35.29 ± 6.56	36.35 ± 5.99	1.275	0.204
C-reactive protein	37.49 ± 43.20	34.22 ± 41.34	-0.588	0.557
Total protein (g/L)	64.83 ± 7.49	65.00 ± 71.11	0.171	0.864
Albumin(g/L)	38.93 ± 5.36	42.83 ± 33.53	1.383	0.168
Serum ferritin(umol/L)	8.75 ± 7.78	8.83 ± 5.20	0.090	0.929
D-dimer (mg/L)	8.18 ± 10.03	9.17 ± 11.02	0.726	0.469
PT activity(%)	104.36 ± 16.31	106.48 ± 17.97	0.950	0.343
INR	1.06 ± 0.12	1.03 ± 0.14	-1.717	0.087
Fibrinogen	3.84 ± 1.17	3.64 ± 1.22	-1.319	0.189
Fibrin degradation product	37.05 ± 38.52	39.02 ± 42.91	0.373	0.710

The continuous value was given as the mean and the standard deviation. Categorical values are given as the number of patients. *TXA* Tranexamic acid, *EACA* Epsilon aminocaproic acid, *BMI* Body mass index = weight/height2, *INR* International normalized ratio

### Blood loss and blood transfusion volume

DBL (164.31 ± 123.98 ml) in the EACA group was significantly lower than that in the TXA group (261.49 ± 190.44 ml) (*p* < 0.0001); PBV, TBL, HBL, and perioperative transfusion were also significantly different between the two groups (*p* > 0.05). The related outcomes are summarized in Table 2.

**Table 2:** Comparison of blood loss and blood transfusion volume

Index	EACA(*n* = 146)	TXA(*n* = 97)	t	*p*
PBV(L)	3.86 ± 0.71	4.01 ± 0.72	1.493	0.137
TBL(ml)	580.92 ± 863.65	746.30 ± 731.14	1.552	0.122
DBL(ml)	164.31 ± 123.98	261.49 ± 190.44	4.819	< 0.0001*
HBL(ml)	625.51 ± 793.47	711.61 ± 750.05	0.847	0.398
Volume of transfusion(ml)	234.25 ± 362.94	223.08 ± 317.59	0.005	0.847
Transfusion (n, 100%)	41(28.1%)	37(38.1%)	2.707	0.100

The continuous value was given as the mean and the standard deviation. Categorical values are given as the number of patients. *PBV* Patient’s blood volume. *TBL* Total blood loss, *DBL* Dominant blood loss, *HBL* Hidden blood loss

^*^The *p*＜0.05，and The differences between the two groups were statistically significant

### Index of routine blood tests, C-reactive protein and coagulation function on the first and fifth days after surgery

The C-reactive protein level (68.53 ± 40.80) was significantly better than that in the TXA group (82.55 ± 49.69) (*p* = 0.022); the red cell width (39.24 ± 15.08 and 35.56.94 ± 16.91) was significantly better than that in the aminohexanic acid group (43.41 ± 11.52, 42.46 ± 12.48) (p < 0.05). However, there were no significant differences in red blood cells, platelets, red blood cell width, hemoglobin, hematocrit, total protein, albumin, serum iron ion, D-dimer, PT activity, international standard ratio, fibrinogen, and fibrin degradation products on days 1 and 5 after surgery (*p* > 0.05). The related outcomes are summarized in Table 3. LOH, operation time, hospital expense, complete weight-bearing time and operation time.

**Table 3:** Comparison of the results of routine blood, C-reactive protein and coagulation tests on day 1 after surgery

Varible	EACA(*n *= 146)	TXA(*n* = 97)	t	*p*
RBC(10^12^/L)	3.27 ± 0.50	3.26 ± 0.58	-0.201	0.841
Platelets(10^12^/L)	261.97 ± 85.16	259.70 ± 90.45	-0.199	0.843
Red blood cell width	43.41 ± 11.52	39.24 ± 15.08	-2.435	0.022
HB	100.19 ± 15.47	100.25 ± 16.52	0.031	0.975
Hematocrit	35.29 ± 6.56	36.35 ± 5.99	-0.642	0.522
C-reactive protein	68.53 ± 40.80	82.55 ± 49.69	2.402	0.022*
Total protein	64.83 ± 7.49	65.00 ± 7.11	0.941	0.348
Albumin	32.48 ± 4.03	32.78 ± 4.32	0.548	0.584
Serum ferritin	4.97 ± 2.81	5.39 ± 4.68	0.878	0.381
D-dimer (mg/L)	6.31 ± 5.01	5.20 ± 4.67	-1.735	0.084
PT activity	94.91 ± 12.66	94.68 ± 16.45	-0.126	0.905
INR	1.11 ± 0.14	1.10 ± 0.17	-0.742	0.459
Fibrinogen	4.56 ± 1.09	4.77 ± 1.25	1.352	0.178
FDP	24.17 ± 20.66	21.11 ± 23.55	-1.068	0.286

The continuous value was given as the mean and the standard deviation. *RBC* Red blood cell, *HB* Hemoglobin, *INR* international normalized ratio, *FDP* Fibrin degradation product

^*^The *p*＜0.05，and The differences between the two groups were statistically significant

**Fig. 3:**
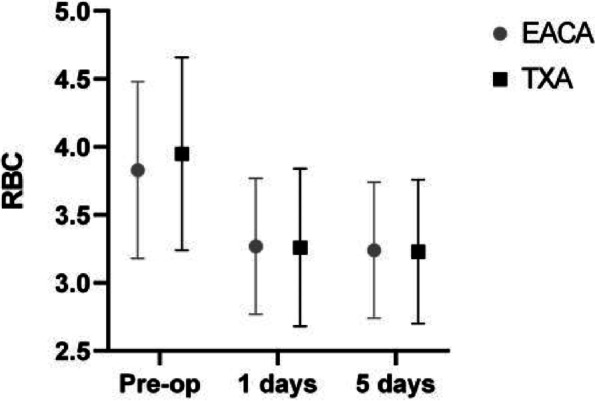
Comparison of the first, postoperative day and fifth postoperative day after RBC surgery

**Fig. 4:**
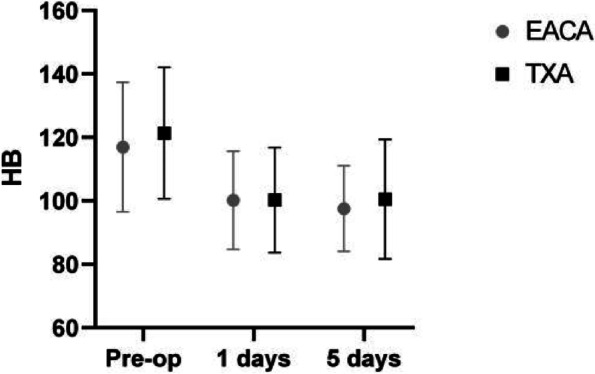
Comparison of the first, postoperative day and fifth postoperative day after HB surgery

There was no significant difference in the length of hospital stay, surgery time, total hospitalization cost or complete weight-bearing time between the two groups (*p* > 0.05). Related outcomes are summarized in Tables 3 and 4. The comparison of red blood cells and hemoglobin before and after surgery is shown in Figs. 3 and 4.

**Table 4:** Comparison of the results of routine blood, C-reactive protein and coagulation tests on day 5 after surgery

Varible	EACA(*n* = 146)	TXA(*n* = 97)	t	*p*
RBC(1012/L)	3.24 ± 0.50	3.23 ± 0.53	-0.097	0.923
Platelets(1012/L)	295.91 ± 92.52	300.23 ± 112.80	0.324	0.746
Red blood cell width	42.46 ± 12.48	36.54 ± 16.91	-3.13	0.004
HB	97.52 ± 13.48	100.52 ± 18.83	1.446	0.178
Hematocrit	30.63 ± 4.48	30.24 ± 4.81	0.388	0.699
C-reactive protein	64.35 ± 43.59	76.32 ± 52.76	1.918	0.056
Total protein	56.20 ± 7.06	57.05 ± 6.72	-0.435	0.664
Albumin	33.30 ± 6.25	33.41 ± 3.85	0.153	0.878
Serum ferritin	6.48 ± 3.84	6.61 ± 3.52	0.277	0.782
D-dimer (mg/L)	5.10 ± 2.66	4.65 ± 2.65	-1.277	0.203
PT activity	98.10 ± 12.98	96.61 ± 16.51	-0.786	0.433
INR	1.11 ± 0.14	1.15 ± 0.63	0.676	0.499
Fibrinogen	4.93 ± 1.15	5.15 ± 1.31	1.345	0.180
FDP	20.43 ± 13.97	18.62 ± 14.24	-0.978	0.329

The continuous value was given as the mean and the standard deviation. *RBC* Red blood cell, *HB* Hemoglobin, *INR* International normalized ratio, *FDP* Fibrin degradation product

### LOH, operation time, hospital expense, complete weight-bearing time and operation time

There was no significant difference in the length of hospital stay, surgery time, total hospitalization cost or complete weight-bearing time between the two groups (*p* > 0.05). The related outcomes are summarized in Table 5.

**Table 5:** Comparison of length of stay, operative time, total hospital expense, and complete weight bearing time

Varible	EACA(*n* = 146)	TXA(*n* = 97)	*t*	*p*
LOH(day)	18.35 ± 5.73	18.91 ± 9.24	0.584	0.560
Operative time (min)	85.61 ± 37.14	85.00 ± 31.86	-0.134	0.894
Expenses∆	44,300.39 ± 13,486.93	43,320.27 ± 16,970.33	-0.500	0.618
Full weight bearing time(day)	73.14 ± 17.98	76.25 ± 16.09	1.378	0.170

The continuous value was given as the mean and the standard deviation. LOH: length of hospital stay

Stay. ∆ Results are presented in Chinese yuan

### Complications

There was no significant difference in thromboembolism, mortality, or recurrent fracture between the two groups. Fifty-nine cases of muscular calf vein thrombosis (MCVT) occurred in 59 patients in the EACA group and 37 patients in the TXA group. DVT occurred in 20 patients in the EACA group and 117 patients in the TXA group. There was PE in the EACA group and 1 case in the TXA group. In addition, one person in the EACA and TXA groups died in the hospital. The number of deaths at 12-month postoperative follow-up was 10 in the EACA group and 6 in the TXA group. Five people in the EACA group had fractures again within 12 months and seven in the TXA group.The related outcomes are summarized in Table 6.

**Table 6:** Complications

Varible	EACA(*n* = 146)	TXA(*n* = 97)	*t*	*p*
Thromboembolic event(n, 100%)				
MCVT	59 (40.41)	38 (39.17)	0.037	0.847
DVT	20 (13.69)	11 (11.34)	0.981	0.612
PE	0 (0.00)	1 (1.03)	2.170	0.338
mortality(n, 100%)				
Intrahospital mortality	1 (0.68)	1 (1.03)	2.170	0.338
Mortality in 12 months after surgery	10 (6.84)	6 (6.18)	0.042	0.838
Internal fixation failure/secondary fractur	5 (3.42)	7 (7.21)	1.785	0.182

*MCVT* Muscular calf vein thrombosis, *DVT* Deep venous thrombosis, *PE* Pulmonary embolism

## Discussion

Elderly patients are characterized by advanced age, many basic diseases, poor compensatory ability of organs and organs, high incidence of complications, low self-care ability, and high difficulty of nursing. In addition, most elderly patients had anemia. Although most intertrochanteric fractures can be treated by minimally invasive surgery, reducing perioperative blood loss is the key to treatment due to massive bleeding. The use of TXA in hip and knee replacement surgery is well established, whereas the use of EACA in intertrochanteric fractures of the femur has rarely been reported, so we performed this study.

TXA can reduce blood loss and transfusion by reversibly blocking fibrinolysis. Since the first application of TXA in total knee arthroplasty in 1995, numerous practices and studies have confirmed that it is effective in reducing perioperative blood loss and transfusion rates in hip and knee arthroplasty without increasing the incidence of deep vein thrombosis lower extremity [18]. TXA has been widely used in orthopedic surgery [19, 20]. EACA can inhibit the activator of plasminogen so that plasminogen cannot be converted into plasmin and then inhibit the dissolution of protein. It is suitable for the prevention and treatment of bleeding caused by hyperfibrinolysis. Earlier it was widely used in cardiac surgery and only recently in elective orthopedic hip and knee arthroplasty [21]. Although the efficacy and safety of EACA have been clinically proven [22], there are fewer studies on TXA. This study is a retrospective cohort study comparing the hemostatic effect and safety of the above two drugs in elderly patients with femoral intertrochanteric fracture during the perioperative period, n the hope of providing a theoretical basis for clinical decisions on drug use.

The hemostatic effects of two drugs: TXA and EACA, are two drugs commonly used in clinical orthopedic surgery. Previous studies have shown that the mechanisms of action of these two drugs are similar, but few studies have conducted a comprehensive and direct comparison of the two drugs [23, 24]. The results of this study showed that the width of erythrocytes in the tranexamic acid group was significantly better than that in the aminocaproic acid group on postoperative days 1 and 5, with statistically significant differences (*p* < 0.05) WEI et al. [25] conducted a retrospective study showing that tranexamic acid can effectively and safely reduce postoperative blood loss and blood transfusion rates in elderly patients with intertrochanteric fractures. TIAN et al. [26] concluded that tranexamic acid can significantly reduce recessive blood loss in intramedullary fracture surgery in elderly patients. Recently, Churchill et al. [27] reported that EACA and TXA have similar clinical efficacy in reducing blood loss and transfusion requirements during hip replacement, which seems to be more economically advantageous over TXA; therefore, their team suggested a replacement for tranexamic acid in orthopedic surgery. This is consistent with the findings of the authors. The use of aminocaproic acid in the treatment of intertrochanteric fractures of the femur has been reported less frequently. Zhang et al. [28] reported that aminocaproic acid can significantly reduce perioperative blood loss and blood transfusion rates in elderly patients with intertrochanteric fractures treated with PFNA. EACA has been widely used in perioperative hip and knee arthroplasty. Hobbs et al. [29] reported that EACA can reduce blood loss and the blood transfusion rate during primary joint replacement. A randomized controlled trial of 194 patients undergoing total knee arthroplasty showed that the estimated blood loss was higher in the EACA group than in the TXA group when EACA was given intravenously at the start of tourniquet inflation and before the initial incision [30]. This is not entirely consistent with our results. Our results showed that the dominant blood loss in the EACA group (164.31 ± 123.98) was significantly lower than that in the TXA group (261.49 ± 190.44), and the difference was statistically significant (*p* < 0.0001). There was no significant difference in total blood loss, recessive blood loss or perioperative blood transfusion between the two groups (*p* > 0.05). Combining the above literature and the results of this trial, it can be seen that the hemostatic effects of EACA and TXA are similar.

Safety of the two drugs: Thromboembolism is a serious complication that can lead to death after major orthopedic surgery, of which pulmonary embolism and deep venous thrombosis of the lower extremities account for the largest proportion [31]. In general, antifibrinolytic drugs inhibit fibrinolysis by blocking the lysine binding site of plasminogen and therefore increase the potential risk of perioperative thrombosis in surgical patients. However, the safety of EACA and TXA has recently been reported to be very reliable [21, 28] and does not increase the incidence of thromboembolism while stopping bleeding [22]. A study and meta-analysis did not clearly show that the antifibrinolytic drugs TXA or EACA increased the risk of thrombosis [32–34], and the incidence of complications such as surgical site infection, hematoma, deep venous thrombosis and pulmonary embolism was similar between the two drugs. In a report on the safety of EACA and TXA, the overall incidence of pulmonary thromboembolism in these two drugs was 0.15% and 0.19%, respectively, with no significant difference between the two groups [35]. DONG et al. [36] evaluated six studies (756 participants) of aminocaproic acid in total hip arthroplasty and total knee arthroplasty and reported that aminocaproic acid did not increase the risk of thromboembolic events during the perioperative period of joint replacement. Data from the randomized controlled study by YANG et al. showed no significant difference in the length of stay between the amino EACA and TXA groups (*P* > 0.05). In terms of safety, 3 patients in the EACA group and 2 patients in the TXA group experienced MCVT complications, but the difference between the two groups was not statistically significant (*P* > 0.05), and none of them had acute thrombotic complications such as DVT or PE [30].

The results of this study showed no significant difference in the incidence of thromboembolic events between EACA and TXA in perioperative patients with intertrochanteric fractures, and there was no significant difference in long-term effects (complete weight-bearing time, follow-up mortality within 12 months and the incidence of internal fixation failure or secondary fracture). These studies are consistent with the results of our trial, indicating no significant difference between the two drugs in terms of risk of complications such as thromboembolism.

### Limitations of the study

① This trial was a retrospective cohort study, and all data were collected from the database of our hospital. There was some selection bias and recall bias, which may have affected the results. Multicenter, randomized controlled clinical trials should be conducted in the future, and the sample size should be expanded. ② The optimal doses of EACA and TXA have not been determined, so the dose of the drug used in this trial may produce inaccurate results.

## Conclusions

The perioperative efficacy and safety of EACA and TXA in elderly patients with trochanteric fractures were generally similar. EACA can be considered a substitute for TXA, which increases the flexibility of clinical medication. However, the sample size included was limited, so it is necessary to conduct high-quality, large-sample long-term follow-up clinical studies.

## Data Availability

Follow-up regarding the perioperative use of hemostatic agents for intertrochanteric fractures in older femoral patients is not complete, so the dataset analyzed in this study is not publicly available but is available to the corresponding author on reasonable request.

## Abbreviations

EACA: Epsilon-aminocaproic acid
TXA: Tranexamic acid; proximal femoral nail antirotation (PFNA)
BMI: Body mass index
RBC: Red Blood Cell
HB: Hemoglobin
HCT: Hematocrit
INR: International normalized ratio
FDP: Fibrin degradation product
PBV: Patient’s blood volume
TBL: Total blood loss
DBL: Dominant blood loss
HBL: Hidden blood loss
LOH: Length of hospital stay
MCVT: Muscular calf vein thrombosis
DVT: Deep venous thrombosis
PE: Pulmonary embolism
